# A systematic search for positive selection in higher plants (Embryophytes)

**DOI:** 10.1186/1471-2229-6-12

**Published:** 2006-06-19

**Authors:** Christian Roth, David A Liberles

**Affiliations:** 1 Computational Biology Unit, BCCS, University of Bergen, 5020 Bergen, Norway; 2 Department of Biochemistry and Biophysics, Stockholm University, 10691 Stockholm, Sweden; 3 Department of Molecular Biology, University of Wyoming, Dept. 3944, 1000 E. University Avenue, Laramie, WY 82071, USA

## Abstract

**Background:**

Previously, a database characterizing examples of Embryophyte gene family lineages showing evidence of positive selection was reported. Of the gene family trees, 138 Embryophyte branches showed Ka/Ks>>1 and are candidates for functional adaptation. The database and these examples have now been studied in further detail to better understand the molecular basis for plant genome evolution.

**Results:**

Neutral modeling showed an excess of positive and/or negative selection in the database over a neutral expectation centered on the mean Ka/Ks ratio. Out of 673 families with assigned structures, 490 have at least one branch with Ka/Ks >>1 in a region of the protein, enabling a picture of selective pressures delineated by protein structure. Most gene families allowed reconstruction back to the last common ancestor of flowering plants (Magnoliophytes) without saturation of 4- fold degenerate codon position. Positive selection occurred in a wide variety of gene families with different functions, including in the self incompatibility locus, in defense against pathogens, in embryogenesis, in cold acclimation, and in electrontransport. Structurally, selective pressures were similar between alpha-helices and beta- sheets, but were less negative and more variant on the surface and away from the hydrophobic core.

**Conclusion:**

Positive selection was detected statistically significantly in a small and nonrandom minority of gene families in a systematic analysis of embryophyte gene families. More sensitive methods increased the level of positive selection that was detected and presented a structural basis for the role of positive selection in plant genomes.

## Background

The search for lineage- specific genes and phenotypically important lineage- specific evolution has intensified with the increasing number of genome sequences, driven through population genetic (intra- specific) analysis of SNPs and through inter- specific molecular evolutionary analysis [1]. Gene duplication has classically been viewed as a source of evolutionary innovation [2]. Differing views have emerged as to the role of gene duplication in driving evolutionary novelty, including concerted evolution in tandemly repetitive families, mediating genetic robustness, subfunctionalization, and neofunctionalization de novo or involving pre-adaptation [3-7]. In both paralogs (emerging from lineage- specific gene duplication events) and in orthologs, phenotypically important changescan emerge along a specific lineage via changes in gene expression, in alternative splicing, in coding sequence evolution, or through a host of other mechanisms (see [8] for a discussion). This study will focus mostly on coding sequence evolutionary dynamics.

Amino acid substitutions in a protein sequence can be advantageous, neutral or deleterious to the organism. There are several links in this process. An individual substitution can effect individual protein folding or function. The individual protein then interacts with other molecules in the execution of cellular and organismal functions. These functions are selectable as they differentially effect the organism's ability to survive, mate, and reproduce in competition with other individuals in the species, as modulated by the effective population size of the species. On top of this, environments can change, changing the structure and optima in the fitness landscapes with time (giving temporal variation to the flatness/ruggedness of the fitness landscape).

Even without environmental change, the nature of this fitness landscape is open for debate. Driven by the clock- like evolution of many gene families, the view that a large number of substitutions are neutral or nearly neutral on a flat fitness landscape emerged ([9], see [10] for a review). An alternative view to account for this is presented through a selectionist interpretation of a rapidly changing fitness landscape driven by strong selective pressures on protein stability and dynamics [11,12]. Evidence for this view comes from the metastability of proteins. However, it has been shown that the metastability of proteins can emerge as a neutral product of the selection process [13]. This has led to yet another hybrid view, where protein sequences take neutral walks through sequence space, punctuated by rarer adaptive changes. The positive selective effects can be dictated by simple compensatory co- variational changes, but can also be explained by true environmental adaptation.

In examining the evolution of individual sites, the molecular clock frequently breaks down. A gamma distribution has been used to model the distribution of rates across sites to characterize the differences in site-specific selective pressure [14]. More recently, it has been proposed that a single gamma distribution may not be adequate and sites shift co-evolutionarily through evolution in a process called heterotachy [15]. The complexity of this process has even led some to believe that biology has no underlying rules or mechanisms and no models are possible (see for example [16]). Ignoring this last view, we seek to obtain an overview of the substitutional process and characterize the positive selection that has been detected in embryophytes to shed light on the evolutionary process.

Several methods have been developed to detect positive selection in protein-encoding genes (for a review, see [17]). One way to evaluate the selective pressures on protein evolution is to compare the rate of synonymous and non-synonymous nucleotide substitutions. Ks is the estimated number of synonymous changes per synonymous site and corresponds to the rate of amino acid- neutral evolution. Ka, on the other hand, is the number of non- synonymous substitutions per non-synonymous site. Under neutral protein- level evolution Ka should be equal to Ks and hence the ratio Ka/Ks = 1. Deviations from this mark selective pressures at the protein level. It should be noted that Ks itself can be under selective pressures that do not act on Ka, like selection for optimal expression level based upon codon use and tRNA concentration [18]. However, this effect is not expected to generate a large false positive rate in the use of Ka/Ks to detect positive selection.

A Ka/Ks ratio < 1 indicates negative (purifying) selection. The criterion for positive (adaptive) selection is Ka/Ks>1. This is a quite stringent criterion of positive selection, when averaged over an entire gene and is likely to miss certain cases where positive selection is operating (even with more powerful approaches [19]). The method used to calculate the Ka/Ks ratio is averaged over time and so negative selection over the majority of time covering a long branch can mask a short period of positive diversifying selection. Similarly, the method averages over all sites, including those in the hydrophobic core that are frequently under very negative selective pressure to retain a folded scaffold. To avoid that problem, in a straight -forward approach, one can use a sliding window to detect selection (one- dimensional window analysis [20-22]). This approach is designed to detect selective sweeps, where additional mutations hitchhike to fixation together with a positively selected residue. On the other hand, this method will miss strong selective pressures acting on individual regions of a protein driven structurally or functionally. Therefore one can take advantage of a known three- dimensional structure of the protein of interest and group sites according to their proximity in the structure, estimating Ka/Ks in different regions [23,24]. This 3D windowing method works by sliding a sphere of defined radius through a three dimensional structure and calculating Ka and Ks within each sphere for each branch of a three dimensional structure. If the sequence divergence within a family is small, it can generally be assumed that the structure will have changed little over the course of evolution within the family and homology model generation is not necessary. In that case, a solved 3D structure is only needed for one member of a close gene family.

The analysis of the evolution of plant gene families has been limited to a few selected cases where adaptive evolution has been suspected: the self- incompatibility locus proteins in Solanaceae [20], S- phase kinase-associated protein 1 (SKP1) [25], trypsin inhibitors [26,27], the anthocyanin pathway [28,29], and chitinase genes [30]. Other studies focused on specialized genera and discovered adaptive evolution in, for example, cytochrome c oxidase of bladderworts [31]. The release of the fullgenome sequences of *Arabidopsis thaliana *(thale cress; Arabidopsis) [32] and *Oryza sativa *(rice) [33,34] brought a whole new dimension into the field, including the study of gene and genome dynamics [35,36]. Comparing a selection of *Arabidopsis lyrata *(lyrate rocketcress) ESTs with the whole genome sequences from Arabidopsis, Barrier and co- workers [37] discovered that 14 out of 304 orthologous genes revealed signs of adaptive evolution.

Analyzing the data in The Adaptive Evolution Database (TAED) [38], we studied 87 protein families with at least one branch showing a Ka/Ks>>1. Further analysis provided both a characterization of selective pressures and substitution rates, including as dictated by structure using the 3D windowing approach described above.

## Results

**Figure 1:**
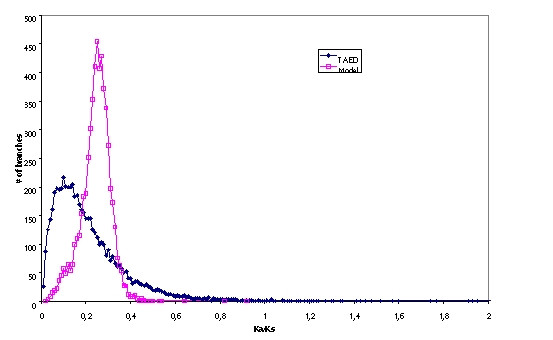
The distribution of Ka/Ks values calculated from TAED and from a null model derived from gene family analysis parameters, as measured with PAML [62], and re- simulated in the absence of positive selection, is shown. An equal number of branches is represented for each dataset. An excess of positive and/or negative selection is observed from the comparative genomic data.

From 138,662 Embryophyte genes from GenBank release 136, 4,216 gene families were built in TAED. The ratios of non- synonymous to synonymous nucleotide substitution rates (Ka/Ks) cover a range from 0.00 to 7.21 per branch with an average value of 0.21 ± 0.04 (Figure 1). Modeling shows that the database contains more negative and/or positive selection than the neutral expectation about the mean Ka/Ks ratio (itself a signal of negative selection). After manual curation of the automated results, 87 families showed Ka/Ks>>1. Of these, 53 families were pairs of putative proteins or proteins of unknown function. Most of these pairs originated from the genome annotations of Arabidopsis and rice. Although most of these pairs originated from the above genome annotations, they were all confirmed by an EST in at least one species (and conservation across species without introduction of spurious stop codons) and therefore were presumed to be real rather than annotation artifacts. 41 of the 53 pairs were even confirmed by EST detection in at least two species. Future embryophyte gene and genome sequencing in these families will create a fuller picture of these evolutionary events.

8 sequence pairs and 26 families with 3 or more sequences of 'known' function showed Ka/Ks rate ratios significantly greater than 1 (Table 1). The highest values were recorded for a ferredoxin in the sequence pair *Zea mays *(maize) and rice (7.21 ± 0.47) and a pair of mannose binding lectins in rice and *Hordeum vulgare *(barley; 5.93 ± 0.28).

**Figure 2:**
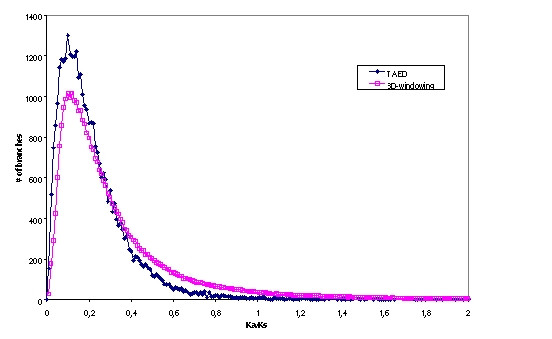
The distribution of Ka/Ks values calculated from TAED using a 3D windowing (structurally- delineated) approach is shown overlaid on the whole gene averaged dataset from Figure 1. An equal number of branches is represented for each dataset. An excess of positive (and negative) selection is detected in the 3D windowing approach compared with the whole gene averaged approach (see the main text for an expanded discussion of this).

We also calculated Ka/Ks rate ratios for families where a structure existed in PDB at a maximal distance of 70 PAM units. The values in the 10Å windows calculated cover a range from 0.00 to 27.46 with an average value of 0.35 ± 0.20 (Figure 2). Out of 673 families, 490 had at least one branch with Ka/Ks>>1.

**Figure 3:**
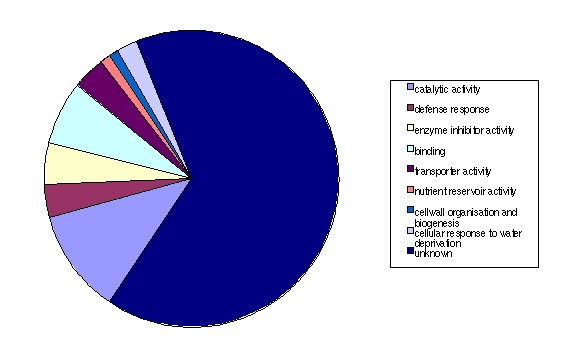
GO annotations for the gene families where positive selection  was detected using whole gene averaging are shown. The text describes these GO annotations normalized by the total number of gene family  branches.

**Figure 4:**
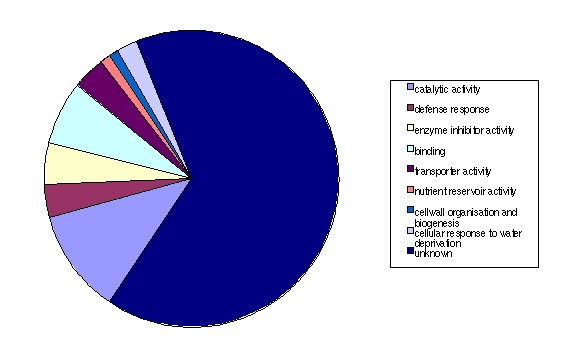
GO annotations for the gene families where positive selection  was detected using 3D windowing are shown. Compared with Figure 3, the  two methods detect different distributions of functions, with an  expanded number of positively selected proteins identified with 3D  windowing. The text describes these GO annotations normalized by the  total number of gene family branches.

The gene families with high Ka/Ks values covered a wide range of cellular functions, including at the self incompatibility locus and at shoot elongation (Figure 3). In the global analysis more than 65% of the families under positive selection were of unknown function. 11% had a catalytic activity and 6% were involved in some kind of binding (DNA or protein). When the large number of proteins of unknown function were excluded from GO annotation categorization, enzyme inhibitors, and genes with nutrient reservoir activity, cell wall organization and biosynthesis, and cellular response to water deprivation functions were over- represented in the high Ka/Ks dataset. When 3D windowing was used (Figure 4), then proteins with catalytic activity, and those involved in protein biosynthesis, development, intracellular transport, RNA/DNA metabolism, and organelle organization and biosynthesis joined the list of over- represented GO terms. Some examples of defense – related proteins were detected, although surprisingly, defense- related proteins in general were not significantly over- represented in the high Ka/Ks dataset.

Within the self incompatibility locus, several RNases seem to have been under positive selection. In Solanaceae, this family has 25 branches with values significantly above 1. In Rosaceae four high values occurred on branches leading to *Pyrus pyrifolia *(sha li) (1.18 ± 0.02), to *Crataegus monogyna *(hawthorn) (1.20 ± 0.02 and 1.10 ± 0.01), and to a clade containing *Malus x domestica *(apple), *Crataegus monogyna *and *Sorbus aucuparia *(rowan) (1.04 ± 0.01). A family of cysteine rich S- locus proteins [39] from Brassica species had 11 significant branches. An S- locus F- box protein in *Prunus mume *(Japanese apricot) showed allelic diversity [40] and we found a Ka/Ks value of 1.31 ± 0.01 on the branch to protein b.

Other families under positive selective pressure are found in plant defense mechanisms. In the chitinase III family of Poaceae a branch of 1.08 ± 0.01 leading to rice was observed. The neighboring branch led to a clade containing other rice sequences and *Triticum turgidum *(poulard wheat). The Triticum gene is annotated as xylanase inhibitor. The chitinase A and B genes were found in family 9484 and showed similarly elevated values on branches to maize as previously reported [30].

Two thionin families, proteins involved in resistance against bacterial pathogens, showed evidence of positive selection. In Poaceae, two values occurred between *Avena sativa *(oat) and barley, 1.21 ± 0.02 on the branch to oat, and between the class V thionins and the purothionins in Triticeae (wheat, oat, barley; 1.30 ± 0.03). A flower- specific gamma thionin in Solanaceae showed a value of 1.29 ± 0.03 on a branch leading to *Lycopersicon esculentum *(tomato) and *Capsicum chinense *(bonnet pepper), compared to a clade with *Petunia x hybrida *(garden petunia) and *Nicotiana excelsior *(tobacco).

CCD1 is an EF- hand Ca2+ binding protein which is involved in fast response to pathogen attacks in wheat [41]. The Ka/Ks value for the sequence pair *Triticum aestivum *(bread wheat) and Arabidopsis on the Magnoliophyta node was 1.08 ± 0.08.

Several Late Embryogenesis Abundant proteins with high Ka/Ks rate ratios were also observed. LEA proteins are involved in desiccation tolerance in the drying seed. The highest value was found for proteins from *Glycine max *(soybean) and Arabidopsis in a subfamily of the D113 proteins (1.91 ± 0.08). Another family included proteins probably involved in cold- response from bread wheat and *Cicer arietinum *(chick- pea) at a value of 1.78 ± 0.07. The third candidate for adaptation on LEA proteins was the PACCAD clade of grasses containing e.g. maize [42]. This clade showed a Ka/Ks rate ratio of 1.12 ± 0.01. BudCAR6 (or CAS15) was a protein associated with cold induced freezing tolerance in *Medicago sativa *(alfalfa) [43]. A homologous protein in *Vitis vinifera *(grape) is induced in ripening fruits [44]. The gene pair had a Ka/Ks value of 1.16 ± 0.06.

A trypsin inhibitor family from poplar had two high branches at 1.76 ± 0.02 and 1.06 ± 0.01 leading to *Populus tremuloides *(trembling aspen). Cathepsin D inhibitors (aspartic proteinase inhibitors) are proteins induced by jasmonic acid, i.e. upon wounding of the plant by herbivory. Genes from *Solanum tuberosum *(potato) and *Solanum nigrum *(black nightshade) showed a value of 1.03 ± 0.01 on the branch to potato.

Proteinase IV is a cysteine proteinase with a preference for glycyl bonds from the latex of papya [45] and had a value of 1.05 ± 0.01 in Caricaceae on the branch to *Carica candamarcensis *(mountain papaya).

Other families showing signs of adaptive evolution were found in plant development and intracellular transport. Cytochrome P450 proteins are involved in steroid synthesis and oxygenation reactions. In rice, a cytochrome P450- like protein was separated by a branch with a value of 1.06 ± 0.01 from a clade with Arabidopsis, rice, and *Musa acuminata *(banana). Dof zinc finger transcription factors are plant specific proteins and showed a Ka/Ks value of 1.14 ± 0.06 in the Magnoliophyta pair Arabidopsis and barley. The phytochrome family is involved in light perception and plant development [46]. Within Coniferales, a branch leading to *Cephalotaxus fortunei *(Fortune's plum yew) with a Ka/Ks value of 1.12 ± 0.01 was observed.

Two families of lipid transfer proteins in monocotyledons also showed signs of positive selection. The first family is root specific and had a Ka/Ks ratio of 1.26 ± 0.03 on a branch towards rice. The second family had two branches under positive selection. The first branch within Triticeae had a value of 1.04 ± 0.02 and led to bread wheat and poulard wheat. The second branch is in Poales and led to Poaceae (rice, barley) with a value of 1.43 ± 0.03.

TIM23 is a family of mitochondrial inner membrane proteins involved in the translocation of proteins in to the mitochondrial matrix. There are generally three isoforms in each species. The branch leading to rice isoform 3 showed a Ka/Ks value of 1.12 ± 0.06.

Ferredoxin is a soluble low molecular weight protein that mediates transfer of one electron from a donor to an acceptor. It is involved in a broad spectrum of redox metabolism in plastids and mitochondria. The extreme value of 7.21 ± 0.47 separated the sequence pair of maize and rice.

Beta expansins and xyloglucane endotransglycosylases are proteins involved in shoot elongation. Both genes seem to have been under positive diversifying selection in the monocot *Schedonorus pratensis *(meadow ryegrass). The beta expansins have a high Ka/Ks value of 1.21 ± 0.01 in comparison to bread wheat. And the xyloglucane endotransglycosylase Xet2 has a value of 1.02 ± 0.01 compared to barley.

In the 3D- windowing approach, 49% of the families with nucleotide substitution rate ratios above 1 had catalytic activity and only 10% were of unknown function. Binding activities occurred in 17 % of the families (Figure 4). The largest number of additional functions observed to have undergone positive selection compared to the global sequence analysis, were hydrolases and MADS- box transcription factors. These proteins have defined specific selectable binding pockets.

In Table 2 we show ratios of identical four- fold degenerate codons for the 10 most populated nodes in the embryophyte tree of life in a pairwise analysis. This shows that even without considering phylogeny, the average family can be analyzed back through the last common ancestor of flowering plants (Magnoliophytes).

In examination of general structural constraints on sequence evolution as seen in Table 3, the hydrophobic core has the strongest negative selection, while residues of intermediate solvent accessibility and those on the surface showed less negative selective pressures with larger variances of selective pressure. No significant differences were seen between secondary structural elements (data not shown).

## Discussion

The database underlying the systematic study performed here contains 4216 gene families. In a global sequence analysis, 87 of these families showed at least one branch with a Ka/Ks ratio above 1. Most of the gene families identified to be under positive selective pressure were just sequence pairs. The majority of these pairs came from the genome annotations of the only two fully sequenced genomes of Arabidopsis and rice. This is also the reason why a lot of these pairs were of unknown function. Furthermore, the two genomes were from quite distant species: Arabidopsis is a dicotyledonous plant, while rice a monocotyledonous plant, and they shared a last common ancestor about 135 – 250 Mya [47]. This point in Table 2 did not, on average, show saturation in a pairwise comparison of sequences.

In the future, more embryophyte genomes will be fully sequenced and this will be reflected in the database that was used for this study. This will eliminate the need to include potentially lower quality EST sequences in the analysis to enrich phylogenetic coverage. The number of species with ESTs is currently much higher than the number of species with full cDNA sequences. In addition, the EST database covers a broader spectrum of cellular functions than the fully sequenced known genes. However, it is anticipated that the general picture of embryophyte coding sequence evolution will remain robust as underlying datasets become fuller.

Most of the gene families we found have molecular functions that are known to be under positive selective pressure. The self- incompatibility locus (S- locus) in flowering plants is a highly polymorphic region that inhibits self- fertilization. The genes in this region are under a high diversifying selective pressure, as Clark and Kao already showed for Solanaceae [20]. Mating and sexual selection are classic examples of an evolutionary arms race.

Defense genes were also expected to show up in our analysis, as they are involved in the cellular arms race (another classic example) against pathogens, and different species show resistance to different pathogens. Individual defense gene examples were detected, but this was not general in a statistically significant way. The functions involved among those detected include chitinases, proteinases, proteinase inhibitors, and lectins. The chitinase III family in our database showed a high Ka/Ks branch to a rice gene, other genes in this clade are annotated as xylanase inhibitors. Further analysis on the sequences and especially their biochemical function will have to show if this clade is working as a chitinase or/and as a xylanase inhibitor *in vivo *and what causes this functional change.

Among the defense related genes we can also find the lectins, which are a quite heterogeneous family of carbohydrate binding proteins involved in defense against herbivory and probably cellular signaling [48]. Other lectins are used as storage in leguminous seeds, where they can act as major food allergens in humans.

The results from the 3D- windowing show a quite different pattern of cellular functions under selective pressure than the global analysis (Figures 3 and 4). Some of this is due to functional sampling bias in PDB. However, the increased detection of positive selection using this approach may be linked to regions having undergone true functional change. It has been proposed that many proteins can change function under positive selection using only a small number of residues [49]. Alternatively, it might be taken as evidence for structural co- evolution in a heterotachy model (some structural co-evolution occurs allosterically- see [50] for example). Interestingly, applying a method that searches for evidence of multiple gamma distributions to characterize a gene family does not show a significant correlation between shifting gammas and Ka/Ks>>1 [[51], Pierre Pontarotti, personal communication]. This lack of a significant correlation remains unexplained, as a correlation would be expected if both methods are detecting the same signal of positive diversifying selection leading to functional shifts.

The distribution of non- synonymous to synonymous nucleotide substitution rate ratios in the database from 0.00 to 7.21, with an average of 0.21 ± 0.04 shows a strong negative selection on most branches in our gene families. The simulated sequences created with the same average Ka/Ks ratio has a much narrower distribution, with many fewer examples of Ka/Ks>1 (Figure 1). This indicates an excess of negative and positive selection acting on the embryophyte tree of life. Comparing the data from the 3D- windowing to the global sequence analysis, we see a similar distribution of values in both cases, with some additional positive selection occurring in the 10 Å windows, shifting the average value to 0.35 ± 0.20. These windows tended to correspond with regions of proteins that were more solvent accessible, consistent with the results seen in Table 3. It should be emphasized that the same codon positions can occur in multiple windows and that more windows contain surface positions than windows containing only hydrophobic core residues. In addition to the greater resolution to detect positive (and negative) selection with a greater variance of Ka/Ks ratios, the asymmetrical distribution of windows can explain the increase in the average Ka/Ks value (see [24] for a discussion of the statistical properties of this method).

In general, the 3D windowing approach offers greater power to detect regions where positive selection may have occurred, as proteins evolve new functions in specified regions of proteins (for example binding pockets) while retaining the same fold and hydrophobic core. 3D windowing is especially attuned to detecting the signal from the region undergoing neofunctionalization without averaging the signal together with that from the highly conserved core and folding- critical residues.

The analysis of the 10 most populated nodes for the fraction of identical four- fold degenerate codons in a pair wise comparison of sequences, shows, that saturation is not reached on average nodes as far back as the last common ancestor of flowering plants 135 to 250 million years ago [47]. This allows us to compare the sequences without ancestral sequence reconstruction on most Magnoliophyte families, including the split between Arabidopsis and rice. Further, using reconstructed ancestral sequences has been shown to increase the evolutionary time that can be accessed without saturation, dependent upon the tree topology and articulation, as reflected in individual branch lengths [52]. There is, of course, a large degree of variation between families and this must be evaluated on a family by family basis.

This study has characterized a snap shot of the evolutionary process acting upon higher plant gene families. We find a large degree of non-random variation in the selective process across gene families, including an excess of positive selection in some functions characterized by an evolutionary arms race. Examining evolution in the context of three dimensional structure adds power to the analysis in detecting additional positive selection and displaying the complexity of the selection process. Future work will extend this analysis to enhance our view of both the general evolutionary process and of the functions that are shifting through protein coding gene sequence as closely related species diverge.

## Conclusion

Positive selection was detected in specific gene families along specific lineages providing a set of candidate genes for functional adaptation that warrant further experimental study. Further, this set of genes represented a small but nonrandom and statistically significant part of plant genome evolution. This global picture, in combination with studies at the population genetic level are providing a general picture of the role of selective forces in plants in shaping biodiversity through lineage- specific processes.

## Methods

The TAED database was built as described in Roth et al. [38]. 138,662 embryophyte sequences longer than 10 amino acids from 4,568 species were extracted from GenBank release 136 [53]. After an all-against- all BLAST search [54] global PAM distances were calculated for each hit using Darwin [55]. Families were built by single linkage clustering from sequences annotated as complete, with pairwise PAM distances of 100 PAM units above a relative length threshold. For each family, a multiple sequence alignment was calculated using POA [56]. Phylogenetic trees were estimated by Bayesian inference using MrBayes [57]. A novel soft parsimony approach [58] was used to simultaneously root the trees and map them onto the NCBI taxonomy. Distant families were split based on alignment quality as measured in the percentage of gaps and where the oldest node in the tree was identified as a duplication event rather than a speciation event. This served to increase alignment quality, a possible source of false positive positive selection. For each branch of every phylogenetic tree, Ka/Ks ratios were calculated using a previously established ancestral sequence reconstruction and counting- based approach (see [59-61]) with a Ks lower cut- off of 0.05. Branches were preliminarily considered significant if Ka- V(Ka)>Ks+V(Ks) and at least two non- synonymous substitutions occurred along the branch. This statistical assessment was refined through evaluation with a neutral null hypothesis, as described below. The procedure described above automatically generated the TAED database and hits were manually curated for this study. Manual evaluation of positively selected genes involved examination of the alignments, where in families with branches with Ka/Ks>1 and alignments where some sequences had a high percentage of gaps, the effect of these sequences on the alignment and subsequent calculation of Ka/Ks ratios was evaluated.

PDB- structures were assigned to families at a distance of 70 PAM units. For each family with a structure, a new multiple sequence alignment was calculated included the structure. From this, sequences were threaded through the structure and contact maps with a radius of 10Å were calculated. Then Ka/Ks ratios were determined within these 10Å- windows for each sphere along each branch of every phylogenetic tree [24]. Significant branches were selected as previously described [24].

To compare the results with a null (actually a combination of neutral and negative selection in the absence of positive selection) model, we used Evolver from the PAML package [62]. Families were generated with non-Ka/Ks parameters sampled proportionately from the distribution obtained by codeml (PAML) analysis of all TAED families and with the amino acid distribution derived from TAED as a whole. Trees and branch lengths were randomly selected from the distribution in the database, and Ka/Ks was set to the mean value of the database (0.21). Ka/Ks values were then calculated for these simulated families as described above. The simulated dataset was used to calculate both p- values and an expected false positive rate, given multiple tests (equivalent to the Bonferroni correction multiplied by the number of tests) to assess statistical significance. Many of these whole gene calculations will likely be unambiguous true positives when more sensitive methods that look at sequence subsets (like [24]) are employed. Employing a null model that is based upon positive selection free parameters estimated from comparative genomic data rather than a null model based upon pseudogenes (which these are not) is expected to give a more realistic (less overly conservative) picture of statistical significance, even though this has not traditionally been employed in evolutionary studies.

Saturation of synonymous sites was calculated as the fraction of identical four- fold degenerate codons in a sequence pair if at least five shared codons occur. These pairwise values were projected onto the node of the species tree that joined them from the mapping of the gene family tree to the species tree. From these pairwise values, average gene saturation at every node in a gene family tree was calculated. Then these nodes were projected onto the corresponding node in the embryophyte tree of life. This is different from the along- branch calculation used in TAED to discard saturated datapoints, but is given as an estimate of how far back (on average), pairwise analysis enables estimation of Ks.

367 families in TAED with close structural homologs in PDB were used to categorize general structural properties of sequence evolution. Using DSSP, both the secondary structural elements and solvent accessibility of each residue site were identified [63]. After partitioning residues into secondary structural categories and solvent accessibility categories, Ka/Ks calculation was performed in the 367 families over each branch for each partition and averaged values compared.

To determine which GO terms [64] were over- represented among the gene lineages having undergone positive selection, the functional annotations of the gene family dataset as a whole and the global and tertiary windowing high Ka/Ks datasets were compared to GO, and gene families assigned to a small number of selected GO terms. Over-representation was determined by examining the ratio of GO term frequency per positively selected branch to GO term frequency in the total set of test branches in the TAED database.

## Authors' contributions

Both CR and DAL contributed in significant ways to the design and execution of this study as well as the writing of this paper.

## Figures and Tables

**Table 1:** A list of Embryophyte gene families with high Ka/Ks values detected using whole gene averaging is shown. The p- value and expected rate of false positive detection given multiple tests per detected branch hypothesis (Bon*) are shown as a measure of statistical significance. Statistical significance is measured against simulated proteins evolved according to measured parameters rather than against evolved pseudogenes evolving neutrally at the amino acid level (as is sometimes used as a null model in evolutionary studies). The null distribution is therefore centered at 0.21 rather than 1.0.

Fam- Nr.	Description	Size	Ka	Ks	Ka/Ks	p- value	Bon*
136	Self incompatibility protein/S8-RNase	70	0.09	0.07	1.18 ± 0.02	<10e - 4	<0.5
			0.07	0.07	1.04 ± 0.01	<10e - 4	<0.5
			0.07	0.06	1.20 ± 0.02	<10e - 4	<0.5
			0.09	0.08	1.10 ± 0.05	<10e - 4	<0.5
172	chitinase III	60	0.15	0.14	1.08 ± 0.01	<10e - 4	<1
218	Cystein proteinase IV	106	0.16	0.15	1.05 ± 0.01	<10e - 4	1
266	Xet2	58	0.10	0.10	1.02 ± 0.01	<10e - 4	<1
401	phytochrome A	637	0.06	0.05	1.12 ± 0.01	<10e - 4	<0.5
667	flowerspecific gamma thionin	7	0.17	0.13	1.29 ± 0.03	<10e - 4	<0.5
678	Metallocarboxypeptidase inhibitor	3	0.09	0.08	1.12 ± 0.03	<10e - 3	<0.5
943	serine/threonine kinase	11	0.11	0.07	1.57 ± 0.02	<10e - 5	<0.5
1138	cytochrome P450 like	26	0.23	0.22	1.06 ± 0.01	<10e - 4	<0.5
1833	pollen coat Protein	6	0.28	0.18	1.52 ± 0.06	<10e - 4	<0.1
			0.32	0.20	1.63 ± 0.05	<10e - 4	<0.1
2453	mannose binding lectin	2	0.58	0.10	5.93 ± 0.28	<10e - 4	<0.5
2461	lectin/alpha -amylase inhibitor	98	-	-	12 values	-	-
2625	putative lipid transfer protein	24	0.26	0.21	1.26 ± 0.03	<10e - 4	<0.5
2687	R- protein	11	0.08	0.05	1.60 ± 0.04	<10e - 4	<0.5
3078	Ferredoxin	2	0.60	0.08	7.21 ± 0.47	<10e - 4	<0.5
3182	AsThi5	13	0.12	0.10	1.21 ± 0.02	<10e - 4	<0.5
			0.24	0.18	1.30 ± 0.03	<10e - 4	<0.5
3384	lipid transfer protein	96	0.09	0.08	1.04 ± 0.02	<10e - 4	<1.0
			0.16	0.09	1.43 ± 0.03	<10e - 4	<0.5
3677	Dof zinc finger transcription factor	2	0.17	0.15	1.14 ± 0.06	<10e - 5	<0.5
4479	S- locus Protein	16	-	-	11 values	-	-
5925	CCD1	2	0.35	0.33	1.08 ± 0.08	<10e - 4	<1.0
6716	beta- type phaseolin	13	0.07	0.06	1.21 ± 0.01	<10e - 5	<0.5
7274	Cystatin proteinase inhibitor	20	0.10	0.10	1.05 ± 0.02	<10e - 4	<1.0
7671	Beta expansins	31	0.18	0.15	1.21 ± 0.01	<10e - 5	<0.5
8102	Cathepsin D inhibitor	3	0.11	0.11	1.03 ± 0.01	<10e - 4	<0.5
8641	self incompatibility ribonuclease	139	-	-	25 values	-	-
9033	S- locus F- box protein	6	0.07	0.05	1.32 ± 0.01	<10e - 5	<0.5
9104	trypsin inhibitor	7	0.09	0.05	1.76 ± 0.02	<10e - 5	<0.5
			0.13	0.12	1.05 ± 0.01	<10e - 4	<0.5
9154	LEA3	8	0.18	0.16	1.12 ± 0.02	<10e - 4	<0.5
16358	TIM23	2	0.34	0.30	1.12 ± 0.07	<10e - 4	<0.5
19762	seed maturation protein PM29	2	0.38	0.20	1.91 ± 0.09	<10e - 4	<0.1
34672	BudCAR6	2	0.29	0.25	1.15 ± 0.06	<10e - 4	<0.5
41180	serine/threonine kinase	28	0.09	0.09	1.10 ± 0.01	<10e - 5	<0.5
42513	LEA, cold response	2	0.44	0.25	1.78 ± 0.07	<10e - 4	<0.5
55717	putative transport protein SEC61	2	0.18	0.16	1.09 ± 0.07	<10e - 3	<1

**Table 2:** The 10 most populated nodes in the Embryophyte tree of life are shown together with the fraction of identical four- fold degenerate codons for all pairwise comparisons linked by that speciation node and also with the number of species in TAED under that node.

Node in Tree Of Life	# of species	fraction of identical four- fold degenerate codons
Embryophyta (plants)	4568	0.31 ± 0.14
Tracheophyta (vascular plants)	4113	0.30 ± 0.18
Euphyllophyta	4096	0.29 ± 0.10
Spermatophyta (seed plants)	4045	0.27 ± 0.10
Magnoliophyta (flowering plants)	3883	0.35 ± 0.12
core eudicotyledons	2651	0.35 ± 0.14
asterids	1069	0.50 ± 0.14
rosids	1257	0.38 ± 0.13
eurosids I	755	0.40 ± 0.12
Poaceae (grass family)	451	0.56 ± 0.16

**Table 3:** After partitioning sites into categories of solvent accessibility for 367 TAED families with solved three dimensional structures, Ka/Ks values were calculated across each family over branches and partitions. A stronger negative selective pressure with less variance is generally observed for the hydrophobic core of proteins.

Solvent Exposed Surface (Å^2^)	Ka/Ks
s < 10	0.16 ± 0.05
10 ≤ s ≤ 30	0.25 ± 0.11
s > 30	0.28 ± 0.14
